# Crystal structure of 3-(2,2-di­bromo­acet­yl)-4-hy­droxy-2*H*-chromen-2-one

**DOI:** 10.1107/S2056989014027947

**Published:** 2015-01-03

**Authors:** Ameni Brahmia, Afef Ghouili, Rached Ben Hassen

**Affiliations:** a Unité de Chimie des Matériaux et de l’Environnement UR11ES25, ISSBAT, Université de Tunis-El Manar, 9, Avenue Dr. Zoheir SAFI, 1006 Tunis, Tunisia

**Keywords:** crystal structure, coumarin derivatives, dibromation, hydrogen bonding, π–π inter­actions

## Abstract

The title compound, C_11_H_6_Br_2_O_4_, is a new coumarin derivative obtained from the reaction of 3-acetyl-4-hy­droxy-2*H*-chromen-2-one with bromine in acetic acid. The hy­droxyl group in involved in an intra­molecular O—H⋯O hydrogen bond. In the crystal, π–π inter­actions between the rings of the bicycle [inter­centroid distances = 3.498 (2) and 3.539 (2) Å] pack mol­ecules into stacks along the *b* axis, and weak inter­molecular C—H⋯O hydrogen bonds further link these stacks into layers parallel to the *ab* plane.

## Abstract 2

In a new coumarin derivative obtained from the reaction of 3-acetyl-4-hy­droxy-2*H*-chromen-2-one with bromine in acetic acid, the hy­droxy group in involved in an intra­molecular O—H⋯O hydrogen bond. In the crystal, π–π inter­actions between the rings of the bicycle pack mol­ecules into stacks along the *b* axis.

## Chemical context   

3-Acetyl-4-hy­droxy-2*H*-chromen-2-one is one of the well-known 3-substituted-4-hy­droxy­coumarins, which form a class of fused-ring heterocycles and occur widely among natural products. Several natural products with the coumarinic moiety exhibit inter­esting biological properties such as anti-oxidant and anti­bacterial (Kayser & Kolodziej, 1997 ▸). They also possess pharmacological activities including anti-inflammatory (Mahidol *et al.*, 2004 ▸), anti­cancer (Wang *et al.*, 2002 ▸) and inhibition of platelet aggregation (Cravotto *et al.*, 2001 ▸). These derivatives are very susceptible to electrophilic substitutions (Dou *et al.*, 1969 ▸); their reaction with bromine can give rise to several compounds used as inter­mediate products which are susceptible to inter­esting substitutions (Takase *et al.*, 1971 ▸) in a wide range of organic syntheses. The bromination of these compounds increases their anti­convulsant activity (Dimmock *et al.*, 2000 ▸), which gives them pharmacological importance. Thus, as part of a study of the effects of substituents on the crystal structures of 3-acetyl-4-hy­droxy­coumarins (Traven *et al.*, 2000 ▸), the structure of 3-(2,2-di­bromo­acet­yl)-4-hy­droxy-2*H*-chromen-2-one, (I), has been determined.
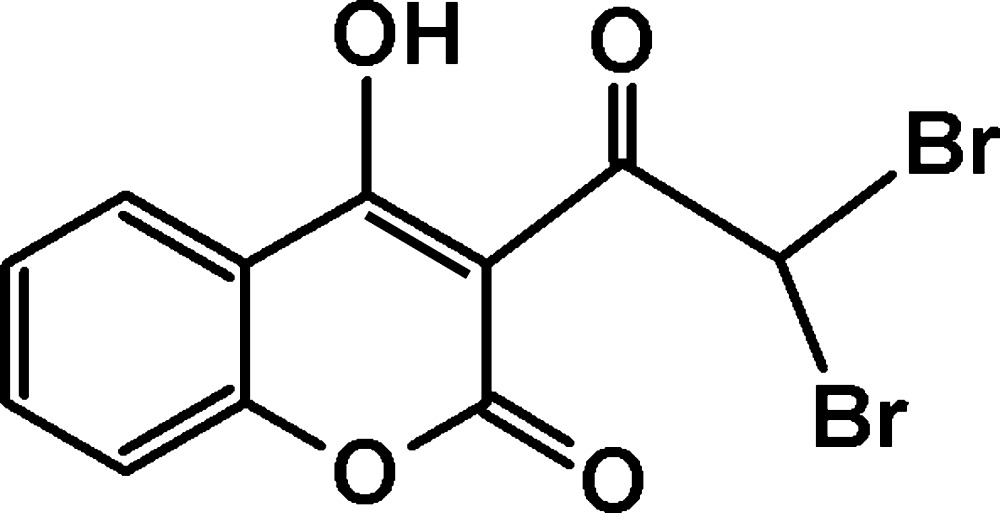


## Structural commentary   

**Figure 1:**
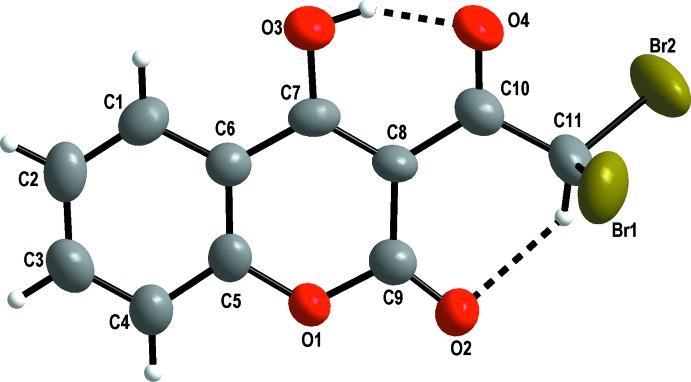
The mol­ecular structure of (I), showing the atom-numbering scheme. Displacement ellipsoids are drawn at the 50% probability level. Intra­molecular hydrogen bonds are shown as dashed lines.

In the title compound (Fig. 1 ▸), the hy­droxy group is involved in formation of an intra­molecular O—H⋯O hydrogen bond (Table 1 ▸). In fact, the O3—H5 distance of 0.94 (7) Å has decreased from 1.02 (3) Å, observed in the starting reagent 3-acetyl-4-hy­droxy-2*H*-chromen-2-one (Lyssenko & Anti­pin, 2001 ▸). The H5⋯O4 distance of 1.65 (7) Å is elongated compared with its value in the parent compound [1.45 (3) Å], and the O3—H5⋯O4 angle of 147 (6)° is significantly smaller than that found for the starting reagent [161 (2)°]. This trend has already been observed in the fluorinated compound 2-di­fluoro­acetyl-1,3-cyclo­hexa­dione (Grieco *et al.*, 2011 ▸), in which the O3—H5 and H5⋯O4 distances are even more affected (0.908 and 1.658 Å respectively). These observations can be easily understood from the point of view of the strong attractive effect of the halogen atoms due to their high electronegativities. All these geometrical parameters are in good agreement with the significant attractor effect of the halogen atoms, which affects the lone pairs of the oxygen atom O4, leading to a decrease of the attractor effect of O4 in the H5⋯O4 hydrogen bond and, consequently, an increase in the H5⋯O4 distance.

The C—C and C—O bond lengths in (I) correspond well to those observed in the parent compound, so they are not affected by the α-ketodibromation except for C10—C11 [1.523 (9) Å] which is elongated compared to the distance in the starting reagent [1.485 (2) Å; Lyssenko & Anti­pin, 2001 ▸). This trend had previously been observed in the similar structure of 2-di­fluoro­acetyl-1,3-cyclo­hexa­dione (Grieco *et al.*, 2011 ▸), in which the difluoration reaction affects the C10—C11 distance [1.529 (2) Å].

## Supra­molecular features   

**Figure 2:**
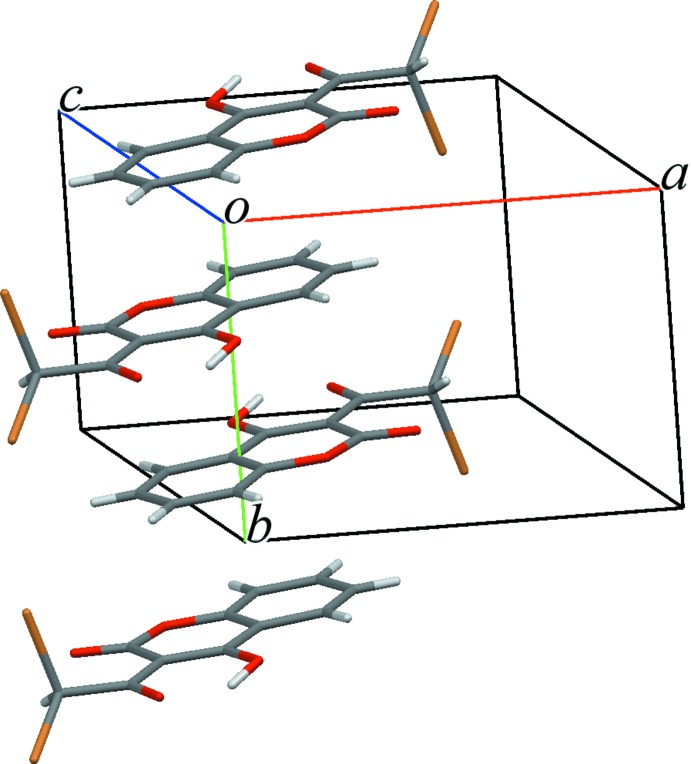
A portion of the crystal packing showing one stack of mol­ecules parallel to the *b* axis.

**Figure 3:**
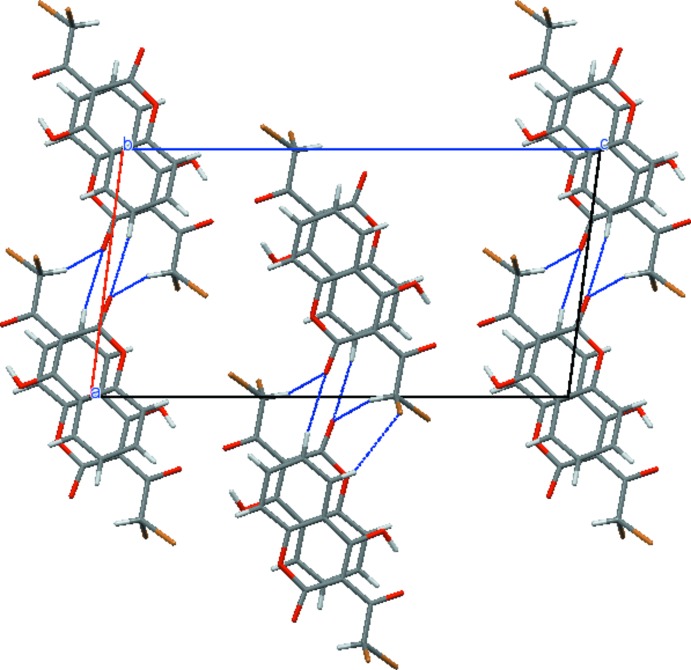
The crystal packing, viewed down the *b* axis, showing the inter­molecular C—H⋯O hydrogen bonds as thin blue lines.

In the crystal structure of (I), the mol­ecules are assembled in a head-to-tail overlapping manner as a result of the π–π inter­actions between the benzene and lactone rings of neighbouring mol­ecules (Table 2 ▸) into stacks along the *b*-axis direction (Fig. 2 ▸). The observed stacking arrangement can be considered as a balance between van der Waals dispersion and repulsion inter­actions, and electrostatic inter­actions between two rings of opposed polarity – the benzene ring (high electron density) and the lactone ring (low electron density) (Hunter & Sanders, 1990 ▸). Weak inter­molecular C—H⋯O hydrogen bonds (Table 2 ▸) further link these stacks into layers parallel to the *ab* plane (Fig. 3 ▸).

## Synthesis and crystallization   

**Figure 4:**
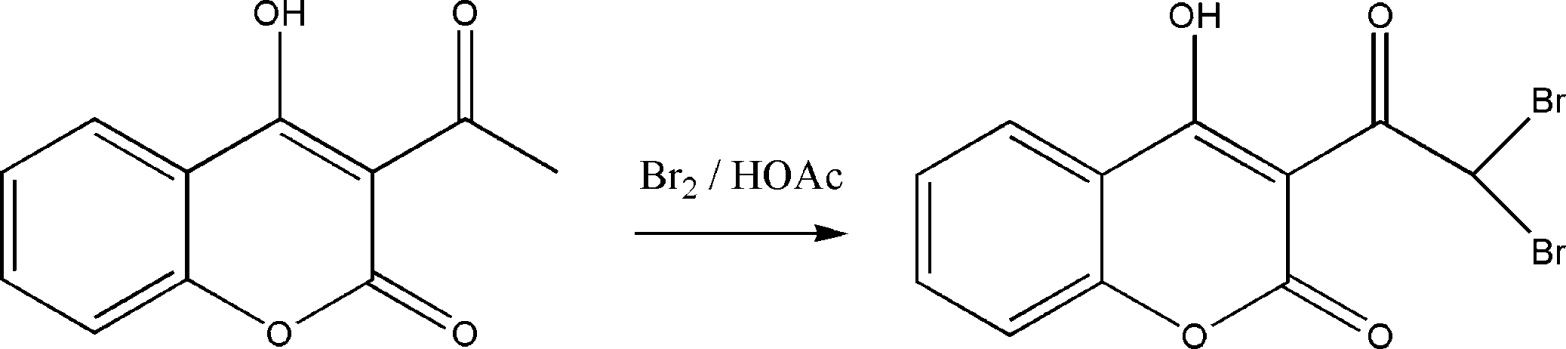
The synthetic route for (I).

An excess amount of bromine dissolved in acetic acid was added dropwise to a solution of 3-acetyl-4-hy­droxy-2*H*-chromen-2-one in acetic acid (Fig. 4 ▸). During the reaction, the dropwise addition was made after every disappearance of the brown colour of the bromine. The reaction mixture was maintained under stirring at 373 K until the bromine colour persisted. The resulting solution was left to crystallize at room temperature to obtain transparent crystals of a light-yellow colour. Yield: 70%; m.p. = 375 K.

## Refinement   

Crystal data, data collection and structure refinement details are summarized in Table 3 ▸. The hy­droxy atom H5 was located from an electron density difference map and freely refined. C-bound H atoms were fixed geometrically (C—H = 0.93 or 0.98 Å) and refined as riding, with *U*_iso_(H) set to 1.2*U*_eq_ of the parent atom.

## Supplementary Material

Crystal structure: contains datablock(s) I. DOI: 10.1107/S2056989014027947/cv5480sup1.cif

Structure factors: contains datablock(s) I. DOI: 10.1107/S2056989014027947/cv5480Isup2.hkl

Supporting information file. DOI: 10.1107/S2056989014027947/cv5480Isup3.cml

CCDC reference: 1040655

Additional supporting information:  crystallographic information; 3D view; checkCIF report

## Figures and Tables

**Table 1:** Hydrogen-bond geometry (, )

*D*H*A*	*D*H	H*A*	*D* *A*	*D*H*A*
O3H5O4	0.94(7)	1.65(7)	2.489(6)	147(6)
C11H11O2	0.98	2.12	2.793(7)	125
C11H11O2^i^	0.98	2.51	3.362(8)	146
C2H2O2^ii^	0.93	2.62	3.458(8)	151

Symmetry codes: (i) 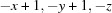; (ii) 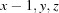.

**Table 2:** Details of interactions: intercentroid distances () *Cg*1 and *Cg*2 are centroids of the C1C6 and O1/C5C9 rings, respectively.

*Cg*1*Cg*2^i^	3.498(7)
*Cg*1*Cg*2^ii^	3.539(7)

Symmetry codes: (i) *x*, *y*+2, *z*; (ii) *x*, *y*+1, *z*.

**Table 3:** Experimental details

Crystal data	
Chemical formula	C_11_H_6_Br_2_O_4_
*M*_r_	361.98
Crystal system, space group	Monoclinic, *P*2_1_/*n*
Temperature (K)	296
*a*, *b*, *c* ()	9.399(4), 6.916(3), 17.967(7)
()	97.37(3)
*V* (^3^)	1158.4(8)
*Z*	4
Radiation type	Mo *K*
(mm^1^)	7.00
Crystal size (mm)	0.15 0.12 0.10

Data collection	
Diffractometer	Bruker *SMART* CCD area detector
Absorption correction	For a sphere (*WinGX*; Farrugia, 2012 ▸)
*T*_min_, *T*_max_	0.58, 0.75
No. of measured, independent and observed [*I* > 2(*I*)] reflections	11685, 3234, 1094
*R*_int_	0.089
(sin /)_max_ (^1^)	0.712

Refinement	
*R*[*F* ^2^ > 2(*F* ^2^)], *wR*(*F* ^2^), *S*	0.057, 0.133, 0.96
No. of reflections	3234
No. of parameters	158
H-atom treatment	H atoms treated by a mixture of independent and constrained refinement
_max_, _min_ (e ^3^)	0.43, 0.48

Computer programs: *SMART* and *SAINT* (Bruker, 2001 ▸), *SHELXS97* and *SHELXL97* (Sheldrick, 2008 ▸), *ORTEP-3 for Windows* and *WinGX* (Farrugia, 2012 ▸).

## supplementary crystallographic information

### Crystal data

**Table:**

C_11_H_6_Br_2_O_4_	*Z* = 4
*M**_r_* = 361.98	*F*(000) = 696
Monoclinic, *P*2_1_/*n*	*D*_x_ = 2.076 Mg m^−^^3^
Hall symbol: -P 2yn	Melting point: 375 K
*a* = 9.399 (4) Å	Mo *K*α radiation, λ = 0.71073 Å
*b* = 6.916 (3) Å	µ = 7.00 mm^−^^1^
*c* = 17.967 (7) Å	*T* = 296 K
β = 97.37 (3)°	Needle, yellow
*V* = 1158.4 (8) Å^3^	0.15 × 0.12 × 0.10 mm

### Data collection

**Table:**

Bruker SMART CCD area-detector diffractometer	3234 independent reflections
Radiation source: fine-focus sealed tube	1094 reflections with *I* > 2σ(*I*)
Graphite monochromator	*R*_int_ = 0.089
φ and ω scans	θ_max_ = 30.4°, θ_min_ = 2.3°
Absorption correction: for a sphere (*WinGX*; Farrugia, 2012)	*h* = −10→13
*T*_min_ = 0.58, *T*_max_ = 0.75	*k* = −6→8
11685 measured reflections	*l* = −25→25

### Refinement

**Table:**

Refinement on *F*^2^	Primary atom site location: structure-invariant direct methods
Least-squares matrix: full	Secondary atom site location: difference Fourier map
*R*[*F*^2^ > 2σ(*F*^2^)] = 0.057	Hydrogen site location: inferred from neighbouring sites
*wR*(*F*^2^) = 0.133	H atoms treated by a mixture of independent and constrained refinement
*S* = 0.96	*w* = 1/[σ^2^(*F*_o_^2^) + (0.0409*P*)^2^ + 0.259*P*] where *P* = (*F*_o_^2^ + 2*F*_c_^2^)/3
3234 reflections	(Δ/σ)_max_ < 0.001
158 parameters	Δρ_max_ = 0.43 e Å^−^^3^
0 restraints	Δρ_min_ = −0.48 e Å^−^^3^

### Special details

**Table:**

Geometry. All e.s.d.'s (except the e.s.d. in the dihedral angle between two l.s. planes) are estimated using the full covariance matrix. The cell e.s.d.'s are taken into account individually in the estimation of e.s.d.'s in distances, angles and torsion angles; correlations between e.s.d.'s in cell parameters are only used when they are defined by crystal symmetry. An approximate (isotropic) treatment of cell e.s.d.'s is used for estimating e.s.d.'s involving l.s. planes.
Refinement. Refinement of *F*^2^ against ALL reflections. The weighted *R*-factor *wR* and goodness of fit *S* are based on *F*^2^, conventional *R*-factors *R* are based on *F*, with *F* set to zero for negative *F*^2^. The threshold expression of *F*^2^ > σ(*F*^2^) is used only for calculating *R*-factors(gt) *etc*. and is not relevant to the choice of reflections for refinement. *R*-factors based on *F*^2^ are statistically about twice as large as those based on *F*, and *R*- factors based on ALL data will be even larger.

### Fractional atomic coordinates and isotropic or equivalent isotropic displacement parameters (Å^2^)

**Table:**

	*x*	*y*	*z*	*U*_iso_*/*U*_eq_	
Br1	0.56249 (8)	0.89000 (12)	0.15244 (5)	0.0906 (3)	
Br2	0.58984 (10)	0.45607 (13)	0.21230 (4)	0.1029 (4)	
O1	0.1772 (4)	0.7341 (5)	−0.05777 (19)	0.0525 (10)	
O2	0.4010 (5)	0.6891 (6)	−0.0136 (2)	0.0637 (12)	
O3	0.0411 (5)	0.6852 (6)	0.1484 (2)	0.0639 (12)	
O4	0.2923 (5)	0.6171 (7)	0.2042 (2)	0.0782 (14)	
C1	−0.1597 (7)	0.7718 (8)	0.0249 (3)	0.0552 (16)	
H1	−0.1941	0.7656	0.0711	0.066*	
C2	−0.2515 (7)	0.8099 (8)	−0.0390 (4)	0.0609 (17)	
H2	−0.3490	0.8267	−0.0365	0.073*	
C3	−0.1981 (8)	0.8233 (8)	−0.1075 (3)	0.0590 (17)	
H3	−0.2612	0.8507	−0.1504	0.071*	
C4	−0.0559 (7)	0.7973 (8)	−0.1138 (3)	0.0533 (16)	
H4	−0.0219	0.8061	−0.1601	0.064*	
C5	0.0358 (7)	0.7576 (7)	−0.0489 (3)	0.0453 (15)	
C6	−0.0123 (6)	0.7421 (7)	0.0202 (3)	0.0426 (14)	
C7	0.0922 (7)	0.7009 (8)	0.0839 (3)	0.0476 (15)	
C8	0.2351 (7)	0.6807 (8)	0.0762 (3)	0.0448 (14)	
C9	0.2804 (8)	0.7008 (8)	0.0022 (3)	0.0489 (15)	
C10	0.3352 (7)	0.6382 (8)	0.1427 (3)	0.0576 (17)	
C11	0.4958 (7)	0.6271 (9)	0.1383 (3)	0.0637 (18)	
H11	0.5117	0.5825	0.0883	0.076*	
H5	0.117 (8)	0.628 (9)	0.180 (3)	0.08 (2)*	

### Atomic displacement parameters (Å^2^)

**Table:**

	*U*^11^	*U*^22^	*U*^33^	*U*^12^	*U*^13^	*U*^23^
Br1	0.0551 (5)	0.1001 (6)	0.1139 (7)	−0.0050 (4)	0.0000 (4)	−0.0223 (5)
Br2	0.1112 (8)	0.1317 (8)	0.0614 (5)	0.0584 (6)	−0.0057 (4)	0.0091 (4)
O1	0.044 (3)	0.071 (3)	0.042 (2)	0.005 (2)	0.004 (2)	0.0054 (18)
O2	0.044 (3)	0.093 (3)	0.055 (3)	0.010 (2)	0.008 (2)	0.006 (2)
O3	0.061 (3)	0.088 (3)	0.044 (3)	0.006 (3)	0.012 (2)	−0.003 (2)
O4	0.066 (3)	0.130 (4)	0.037 (2)	0.005 (3)	0.003 (2)	0.001 (2)
C1	0.052 (5)	0.054 (4)	0.062 (4)	−0.001 (3)	0.017 (4)	−0.007 (3)
C2	0.048 (4)	0.054 (4)	0.078 (5)	0.003 (3)	−0.005 (4)	−0.002 (3)
C3	0.062 (5)	0.053 (4)	0.058 (4)	0.002 (3)	−0.006 (4)	0.006 (3)
C4	0.046 (5)	0.054 (4)	0.058 (4)	0.000 (3)	0.001 (3)	0.004 (3)
C5	0.045 (4)	0.035 (4)	0.055 (4)	0.001 (3)	0.003 (3)	0.004 (3)
C6	0.048 (4)	0.036 (4)	0.043 (4)	−0.003 (3)	0.006 (3)	−0.003 (2)
C7	0.059 (5)	0.046 (4)	0.040 (4)	−0.008 (3)	0.016 (3)	−0.004 (3)
C8	0.050 (4)	0.054 (4)	0.031 (3)	0.001 (3)	0.007 (3)	0.000 (3)
C9	0.054 (5)	0.045 (4)	0.048 (4)	0.005 (3)	0.007 (4)	0.000 (3)
C10	0.062 (5)	0.064 (4)	0.045 (4)	0.005 (3)	0.002 (4)	−0.001 (3)
C11	0.058 (5)	0.086 (5)	0.044 (4)	0.011 (4)	−0.006 (3)	−0.004 (3)

### Geometric parameters (Å, º)

**Table:**

Br1—C11	1.930 (6)	C2—H2	0.9300
Br2—C11	1.910 (6)	C3—C4	1.368 (8)
O1—C5	1.368 (6)	C3—H3	0.9300
O1—C9	1.374 (6)	C4—C5	1.386 (7)
O2—C9	1.206 (7)	C4—H4	0.9300
O3—C7	1.315 (6)	C5—C6	1.379 (7)
O3—H5	0.94 (7)	C6—C7	1.438 (7)
O4—C10	1.233 (7)	C7—C8	1.375 (8)
C1—C2	1.370 (7)	C8—C10	1.453 (7)
C1—C6	1.413 (8)	C8—C9	1.454 (8)
C1—H1	0.9300	C10—C11	1.523 (9)
C2—C3	1.391 (8)	C11—H11	0.9800
			
C5—O1—C9	121.8 (5)	C1—C6—C7	123.8 (5)
C7—O3—H5	103 (4)	O3—C7—C8	123.5 (5)
C2—C1—C6	119.7 (6)	O3—C7—C6	115.4 (6)
C2—C1—H1	120.2	C8—C7—C6	121.1 (5)
C6—C1—H1	120.2	C7—C8—C10	118.4 (5)
C1—C2—C3	119.6 (6)	C7—C8—C9	119.1 (5)
C1—C2—H2	120.2	C10—C8—C9	122.5 (6)
C3—C2—H2	120.2	O2—C9—O1	114.7 (5)
C4—C3—C2	122.2 (6)	O2—C9—C8	127.1 (6)
C4—C3—H3	118.9	O1—C9—C8	118.2 (6)
C2—C3—H3	118.9	O4—C10—C8	120.7 (6)
C3—C4—C5	117.7 (6)	O4—C10—C11	118.7 (5)
C3—C4—H4	121.1	C8—C10—C11	120.6 (6)
C5—C4—H4	121.1	C10—C11—Br2	111.6 (4)
O1—C5—C6	122.1 (5)	C10—C11—Br1	104.7 (4)
O1—C5—C4	115.7 (5)	Br2—C11—Br1	112.2 (3)
C6—C5—C4	122.2 (6)	C10—C11—H11	109.4
C5—C6—C1	118.7 (5)	Br2—C11—H11	109.4
C5—C6—C7	117.5 (6)	Br1—C11—H11	109.4

### Hydrogen-bond geometry (Å, º)

**Table:**

*D*—H···*A*	*D*—H	H···*A*	*D*···*A*	*D*—H···*A*
O3—H5···O4	0.94 (7)	1.65 (7)	2.489 (6)	147 (6)
C11—H11···O2	0.98	2.12	2.793 (7)	125
C11—H11···O2^i^	0.98	2.51	3.362 (8)	146
C2—H2···O2^ii^	0.93	2.62	3.458 (8)	151

Symmetry codes: (i) −*x*+1, −*y*+1, −*z*; (ii) *x*−1, *y*, *z*.
